# Patient perceptions and knowledge of corticosteroid injections: A cross-sectional survey study

**DOI:** 10.1371/journal.pone.0344201

**Published:** 2026-03-18

**Authors:** Juliet Chung, Sina Ramtin, Philip Koehler, Stephen Stache, Charles Langman, Brian Hozack, Asif M. Ilyas

**Affiliations:** 1 Wake Forest Baptist Medical Center, Winston-Salem, North Carolina, United States of America; 2 Rothman Orthopaedic Institute at Thomas Jefferson University, Philadelphia, Pennsylvania, United States of America; 3 University Texas at Austin, Austin, Texas, United States of America; 4 Drexel University College of Medicine, Philadelphia, Pennsylvania, United States of America; University of Illinois Medical Center at Chicago: University of Illinois Hospital, UNITED STATES OF AMERICA

## Abstract

**Introduction:**

Although cortisone injections are commonly used, patient understanding of cortisone is variable and often affected by misconceptions. This study explores patient perspectives, identifies misconceptions, and emphasizes the importance of improved patient education to enhance patient-centered care.

**Methods:**

A cross-sectional survey study was conducted from April to August 2024 among patients aged 18 and older presenting to an orthopedic physicians’ office. Patients completed a novel anonymous questionnaire designed to assess their knowledge and perceptions of cortisone injections. A total of 246 patients participated, with responses collected electronically via SurveyMonkey.

**Results:**

Among 246 respondents, individuals with history of prior cortisone infections were significantly more likely to correctly identify its anti-inflammatory role (p < 0.05). Overall, 96% of respondents reported awareness of cortisone injections. However, many learned from personal connections rather than healthcare professionals as their primary source of information Most respondents (87%) reported receiving satisfactory explanations from their physicians, while 13% reported inadequate communication. A majority indicated willingness to receive cortisone injections if clinically indicated (84%). Despite this,concerns regarding safety persist, with 37% associating safety with dosage and injection frequency, and 15% reporting uncertainty about potential risks.

**Conclusion:**

Misconceptions regarding cortisone injections remain prevalent, highlighting gaps in patient understanding. Although most patients recognize the anti-inflammatory effects of cortisone, knowledge deficits persist regarding safe dosing parameters and potential risks. Improved communication between healthcare providers and patients about cortisone’s mechanism of action and safety profile may support more informed clinical decision-making and outcomes.

## Introduction

Corticosteroids are steroid hormones produced in the adrenal gland. Although the terms corticosteroids and cortisone are often used interchangeably, cortisone is a naturally occurring corticosteroid metabolite first discovered in 1929 by Philip Hench, Edward Kendall, and Tadeus Reichstein while investigating the hormones of the adrenal cortex [1]. Clinical application of cortisone began in the 1950s with its use in the treatment of rheumatoid arthritis. Since then, corticosteroids have become a mainstay in the management of inflammatory conditions and has been used in the treatment of nearly 200 disorders [2–4].

In musculoskeletal medicine, cortisone injections are commonly used to manage conditions such as arthritis, bursitis, tendinopathies, back pain, and carpal tunnel syndrome [5,6]. These injections are widely utilized due to their relatively low cost and demonstrated effectiveness in providing short-term pain relief and functional improvement [7]. Cortisone injections are administered by physicians across multiple specialties including orthopedic surgery, rheumatology, radiology, pain management, sports medicine, and primary care [8–10]. Despite their widespread use, patient understanding of cortisone injections remain variable, with persistent misconceptions.

The purpose of this study was to evaluate patient knowledge, perceptions, and beliefs beliefs regarding cortisone injections, with the aim of identifying common misconceptions and informing opportunities for improved patient education to enhance patient-centered care.

## Methods

Institutional review board approval was obtained through Thomas Jefferson University before the initiation of this anonymous cross-sectional survey study. The approval number was iRISID-2023–2687.

From April to August 2024, patients aged 18 years and older presenting for outpatient visits at the Rothman Orthopaedic Institute at Thomas Jefferson University in Philadelphia were invited to participate in this study. The survey was administered in person during routine office visits. After being escorted to a private examination room, informed consent was obtained from eligible patients and they received instructions on how to complete the anonymous questionnaire. Patients completed a novel survey developed by the study investigator to assess patient’s experience, knowledge, and perceptions of cortisone injections.

Participating physicians represented a range of musculoskeletal specialty including orthopedic surgery, physical medicine and rehabilitation, primary care sports medicine, and podiatry. This multidisciplinary approach was used to enhance the generalizability of the findings across patients presenting with diverse orthopedic conditions. Over the 5-month study period, 246 patients consented to participate. All survey responses were collected and stored electronically using SurveyMonkey website (SurveyMonkey Inc., San Mateo, CA), and no identifiable patient information was collected.

Descriptive statistics were used to summarize survey responses. Bivariate analyses were performed using logistic regression to compare respondents with and without history of cortisone injections across multiple outcomes including knowledge of cortisone mechanism perceived safety, concerns regarding cortisone injections, perceived limits on the number of safe injections, and willingness to receive cortisone injections. Statistical significance was defined as a p-value < 0.05.

## Results

A total of 246 patients completed the survey between April to August 2024. Respondents included 147 (60.3%) female, 96 (39.3%) men, and one participant identified as other; sex was not reported by two participants. Age distribution were as follows:12 (4.9%) were 18–29 years old, 66 (26.8%) were 30–49 years old, 102 (41.5%) were 50–69 years old, and 66 (26.8%) were 70–89 years old. Most (82.1%) of respondents reported a college education or higher (Table 1).

**Table 1:** Patient demographics of the survey respondents.

Patient demographics	N (%)
**Sex**	
Female	147 (60.3)
Male	96 (39.3)
Other	1 (0.4)
**Age**	
18-29	12 (4.9)
30-49	66 (26.8)
50-69	102 (41.5)
70-89	66 (26.8)
**Highest Level of Education**	
High School	31 (12.6)
Vocational Training	13 (5.3)
College	112 (45.5)
Graduate School	75 (30.5)
Professional School	15 (6.1)

**Fig 1:**
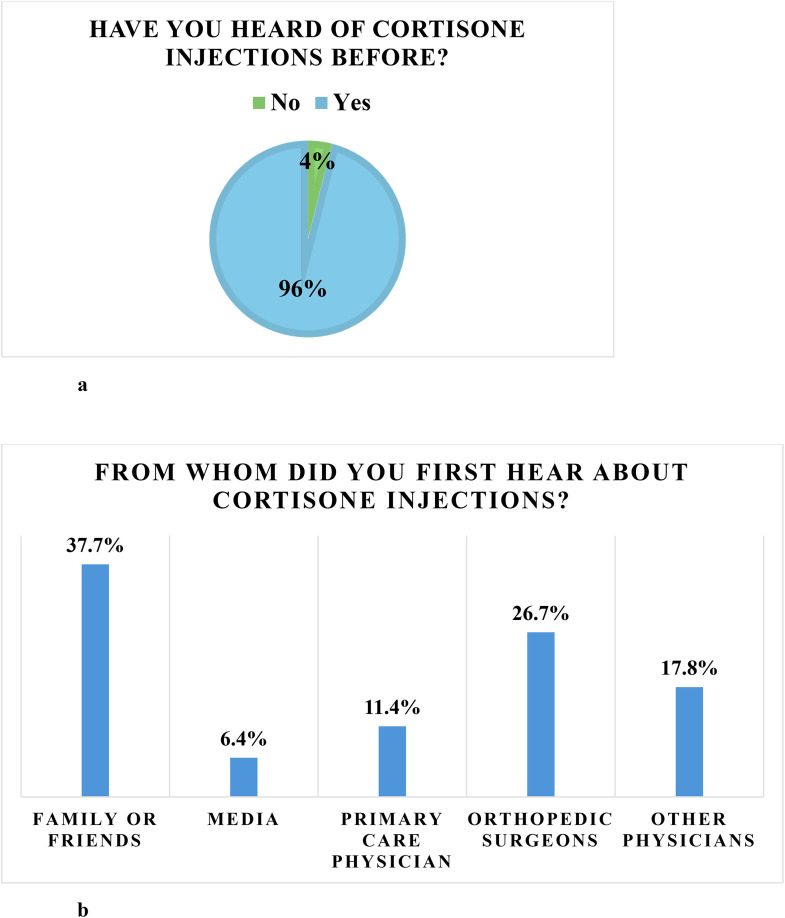
a: Patients who have heard of cortisone injections. 1b: From whom, patients have heard about cortisone injections.

Overall, 236 (95.9%) reported prior awareness of cortisone injections while 10 (4.1%) had not (Fig 1a). Among those aware of cortisone injections, the most commonly reported sources of information were family or friends (37.7%) and orthopedic surgeons (26.7%), followed by other physicians (17.8%), primary care physicians (11.4%), and media sources. (Fig 1b).

**Fig 2:**
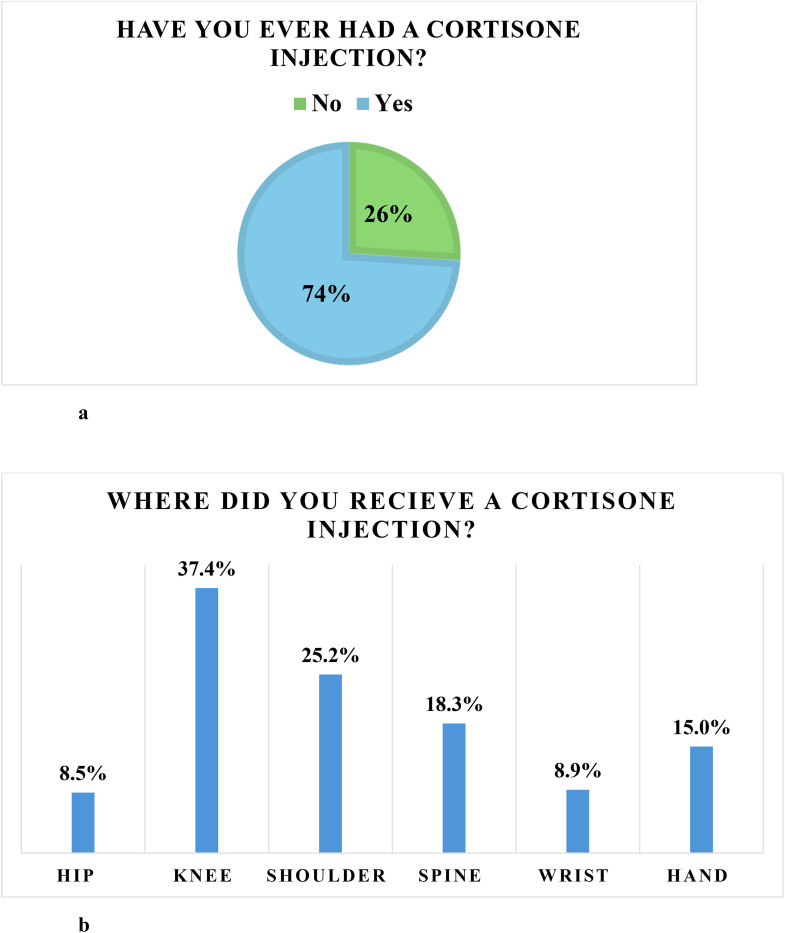
a: Patients who have received a cortisone injection. 2b: In which body region the patient previously received a cortisone injection.

When asked about prior history of cortisone injections, 182 respondents (74%) reported having received at least one injection, while 64 (26%) had not (Fig 2a). Injection sites included the knee (25.2%), shoulder (25.2%), spine (18.3%), hand (15%), wrist (8.9%), and hip (8.5%), with some respondents reporting multiple injection sites. (Fig 2b).

**Fig 3:**
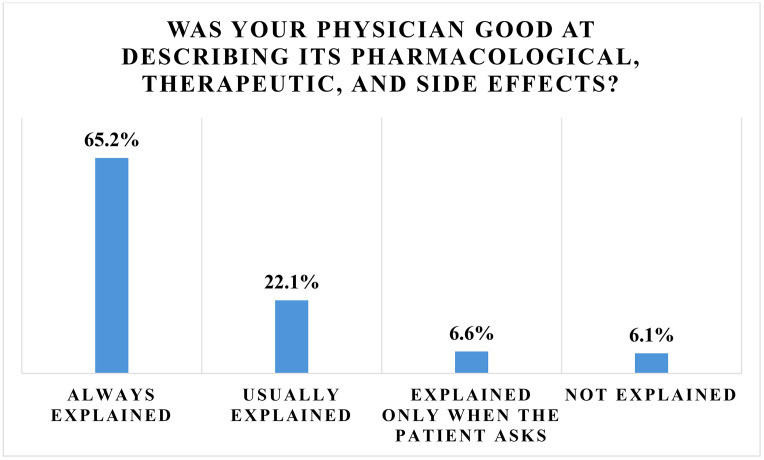
Patients’ opinion about their physician’s ability to explain cortisone injections.

Among respondents who had previously received a cortisone injection118 (65.2%) reported that their physician “always explained” the pharmacologic effects, therapeutic benefits, and potential side effects, while 40 (22.1%) reported that explanations were “usually” provided. Twelve (6.6%) reported explanations were given only upon patient request, and 11 (6.1%) reported that explanations were not provided; one response was left blank (Fig 3).

Regarding knowledge of the maximum number of cortisone injections considered safe in the same anatomic region, 18 respondents (7.3%) selected “one”, 18 (7.3%) selected “two”, and 44 (17.9%) selected “three”, Ninety-two respondents (37.4%) believe there was no particular limit and that safety depended on dosage, while74 (30.1%) reported uncertainty (Table 2a).

**Table 2:** a: Patient understanding of cortisone injections. “From your understanding, how many times can you receive a cortisone injection for the same problem in the same area?”. 2b: Patient understanding of cortisone injections. “What best describes your understanding of cortisone?”. 2c: Patient understanding of cortisone injections. “Do you think cortisone injections are safe?”. 2d: Patient understanding of cortisone injections. “If you think it is unsafe, what do you consider is harmful about cortisone?”. 2e: Patient understanding of cortisone injections. “If you were told you would benefit from it, would you be willing to receive a cortisone injections?”.

2a.	
Max # of Safe Cortisone Injections	N (%)
1 time	18 (7.3)
2 times	18 (7.3)
3 times	44 (17.9)
No particular limit, depends on dosage	92 (37.4)
I don’t know	74 (30.1)
2b.	
Cortisone Mechanism	N (%)
It masks pain	18 (7.3)
It is an anti-inflammatory	173 (70.3)
It numbs pain	27 (11.0)
I don’t know	28 (11.4)
2c.	
Perceived Safety of Cortisone Injections	N (%)
Yes	110 (44.7)
No	7 (2.9)
Depends on dosage and frequency	92 (37.4)
I don’t know	37 (15.0)
2d.	
Concerns of Cortisone Injections	N (%)
It can decrease bone density around the joint	24 (12.8)
It can injure cartilage in the joint	17 (9.1)
It can injure ligaments around the joint	10 (5.4)
I don’t know	85 (45.5)
It is not harmful	51 (27.3)
2e.	
Willingness to Receive Cortisone Injection if Beneficial	N (%)
Yes	206 (84.1)
No	10 (4.1)
I don’t know	29 (11.8)

When asked about the mechanism of cortisone, 173 respondents (70.3%) correctly identified it as an anti-inflammatory agent. Other responses included “it masks pain” (7.3%), “it numbs pain” (11%), and “I don’t know” (11.4%) (Table 2b).

With respect to perceived safety, 110 respondents (44.7%) believed cortisone injections were safe, while 92 (37.4%) reported that safety depended on dosage and injection frequency. Seven respondents (2.9%) believed cortisone injections were unsafe, and 37 (15%) reported uncertainty (Table 2c).

Concerns regarding cortisone injections varied, with 24 (12.8%) citing decreased bone density, 17 (9.1%) citing cartilage injury, 10 (5.4%) citing ligament injury. However, 85 (45.5%) reported uncertainty regarding potential harms and 51 (27.3%) believed cortisone injections were not harmful. Fifty-nine responses were missing for this question (Table 2d).

When asked about willingness to receive a cortisone injection if known to be beneficial, 206 respondents (84.1%) reported willingness, 10 (4.1%) reported unwillingness, and 29 (11.8%) reported uncertainty; one response was missing (Table 2e).

In bivariate analysis, respondents with a history of cortisone injection were significantly less likely to select “I don’t know” when asked about the maximum number of safe cortisone injections, cortisone mechanism, perceived safety, concerns regarding cortisone injections, and willingness to receive cortisone injections (p values <0.05) (Table 3).

**Table 3:** Bivariate logistic regression analysis of responses to cortisone injection knowledge questions categorized as “I don’t know” versus other responses, compared between patients with and without prior cortisone injections. The reference group is patients without injections who answered “I don’t know.”.

	Odds ratio (95% Confidence Interval)	Standard error	P value
**Max # of Safe Cortisone Injections**			
Other Responses	Reference Value		
“I don’t know”	0.17 (0.09 to 0.31)	0.052	**<0.001**
**Cortisone Mechanism**			
Other Responses	Reference Value		
“I don’t know”	0.27 (0.13 to 0.59)	0.11	**<0.001**
**Perceived Safety of Cortisone Injections**			
Other Responses	Reference Value		
“I don’t know”	0.15 (0.07 to 0.32)	0.057	**<0.001**
**Concerns About Cortisone**			
Other Responses	Reference Value		
“I don’t know”	0.36 (0.19 to 0.67)	0.11	**<0.001**
**Willingness to Receive Cortisone Injection if Beneficial**			
Other Responses	Reference Value		
“I don’t know”	0.17 (0.08 to 0.38)	0.069	**<0.001**

Among respondents who did not select “I don’t know,” logistic regression analysis demonstrated a significant association between prior cortisone injection and correct identification of cortisone as an anti-inflammatory agent. Individuals who had received a cortisone injection had higher odds of recognizing cortisone as an anti-inflammatory compared to those who did not (OR = 2.40, 95% CI: 1.01 to 5.72, p = 0.047) (Table 4).

**Table 4:** Bivariate logistic regression analysis comparing each response option to cortisone injection knowledge questions, excluding “I don’t know” responses, between patients with and without prior cortisone injections.

	Odds ratio (95% Confidence Interval)	Standard error	P value
**Max # of Safe Cortisone Injections**			
1 time	Reference Value		
2 times	0.29 (0.03 to 3.14)	0.36	0.31
3 times	0.26 (0.03 to 2.29)	0.29	0.23
No particular limit, depends on dosage	0.33 (0.04 to 2.66)	0.35	0.30
**Cortisone Mechanism**			
It numbs pain	Reference Value		
It is an anti-inflammatory	2.40 (1.01 to 5.72)	1.06	0.047
It masks pain	1.18 (0.34 to 4.12)	0.75	0.80
**Perceived Safety of Cortisone Injections**			
No	Reference Value		
Depends on dosage and frequency	1.54 (0.28 to 8.55)	1.35	0.62
Yes	1.80 (0.33 to 9.95)	1.57	0.50
**Concerns of Cortisone Injections**			
It is not harmful	Reference Value		
It can decrease bone density around the joint	1.89 (0.55 to 6.52)	1.19	0.31
It can injure cartilage in the joint	1.77 (0.44 to 7.09)	1.25	0.42
**Willingness to Receive Cortisone Injection if Beneficial**			
No	Reference Value		
Yes	0.92 (0.19 to 4.49)	0.74	0.92

No statistically significant differences were observed between respondents with and without prior cortisone injections regarding the understanding of the maximum number of cortisone injections, perceived safety, concerns about cortisone injections or willingness to receive cortisone injections if beneficial (Table 4).

## Discussion

Despite universal awareness of cortisone injections among respondents, this study identifies persistent gaps in patient understanding regarding their mechanism of action, safety profile, and appropriate use. Patients with prior experience receiving cortisone injections were more likely to correctly identify their anti-inflammatory effect, suggesting that direct exposure may enhance knowledge acquisition. However, experience alone did not fully mitigate uncertainty, particularly with respect to cumulative dosing, long term safety, and potential adverse effects, indicating that existing patient education may be incomplete or inconsistently delivered.

A key finding is the prominent role of non-medical sources in shaping patient perceptions of cortisone injections. Many respondents reported learning about cortisone through family members or friends rather than healthcare providers. This reliance on informal information networks highlights the broader challenge of misinformation in healthcare and suggests that patient understanding may be influenced by anecdotal experiences rather than evidence-based counseling. Such influences may persist even among patients actively engaged in orthopedic care, emphasizing the need for clinicians to explicitly address and correct misconceptions during clinical encounters.

Although most respondents reported that their physicians provided explanations regarding cortisone injections, uncertainty regarding safety remained common. Many patients perceived cortisone injections as safe under certain conditions while other expressed uncertainty altogether. These findings are consistent with prior literature describing steroid phobia, in which patient exhibit disproportionate concern regarding cortico-steroid associated adverse effects [11–15]. Importantly, these fears may reflect extrapolation from well-known systemic corticosteroid complications rather than risks associated with appropriately administered local injections, highlighting an opportunity for more targeted patient education.

Concerns surrounding cortisone injections may also be influenced by conflicting evidence regarding their long-term efficacy and safety in specific musculoskeletal conditions. Prior studies have demonstrated variable duration of symptom relief, potential risks associated with repeated injections, and limitations related to injection accuracy [16–20]. Such findings may contribute to patient skepticism and reinforces uncertainty regarding the role of corticosteroid injections within broader treatment algorithms. As a result, patient counseling that clearly contextualizes cortisone injections as one component of a comprehensive management strategy instead of a definitive or risk free solution may be valuable.

The high willingness among respondents to receive cortisone injections if clinically beneficial, despite ongoing safety concerns, suggests a complex relationship between patient acceptance and understanding. This apparent contradiction highlights the importance of shared decision making, in which patient values and concerns are integrated with clear, evidence based information. Enhancing communication around mechanism of action, realistic expectations for symptom relief, and individualized risk benefit profiles may help bridge this gap and improve patient confidence in treatment decisions.

This study contributes to the growing literature on patient perceptions of corticosteroid therapy [21] by providing contemporary data from a diverse orthopedic population. The findings reinforce the notion that awareness does not equate to understanding and that patient experience alone is insufficient to ensure accurate knowledge. Addressing these gaps through standardized counseling strategies or patient facing educational materials may represent a modifiable target for improving patient centered care in musculoskeletal medicine.

Several limitations warrant consideration. The study population consisted of patient actively seeking orthopedic care at a single institution, which may limit generalizability. The high educational attainment of respondents may also introduce selection bias and may not reflect populations with lower health literacy. Additionally, reliance on self-reported data introduces the potential for recall bis. This survey was newly developed and not formally validated, which may affect reproducibility. Finally, the cross-sectional design precludes assessment of changes in knowledge over time or casual inference. Future studies should incorporated validated surveys and longitudinal designs to evaluate the effectiveness of targeted educational interventions.

## Conclusion

Despite widespread awareness of cortisone injections, significant gaps remain in patient understanding of their mechanism, safety, and appropriate use. Prior experience improves knowledge but does not fully eliminate misconceptions, which are often reinforced by informal information sources. These findings highlight the need for targeted, clinical led education to correct misunderstandings, enhance patient comprehension. And support informed decision making, ultimately improving treatment outcomes in musculoskeletal care.

## Supporting information

S1 File Cortisone injections survey – physician. Survey instrument used to assess physician perceptions, knowledge, and personal experiences regarding cortisone injections.
(DOCX)

S2 File Cortisone injections survey – patient. Survey instrument used to evaluate patient awareness, understanding, safety perceptions, and willingness to receive cortisone injections.
(DOCX)

S3 File Data. De-identified dataset containing survey responses used for statistical analysis in the present study.
(XLS)
