# Supplementary material for: Patient perceptions and knowledge of corticosteroid injections: A cross-sectional survey study
Source: PLoS One. 2026 Mar 18;21(3):e0344201. doi: 10.1371/journal.pone.0344201 (PMC12998833; doi:10.1371/journal.pone.0344201)
Supplement: S1 File — Survey instrument used to assess physician perceptions, knowledge, and personal experiences regarding cortisone injections. (DOCX) [file pone.0344201.s001.docx]

**Survey of Physician Perception of “Cortisone” Injections Questionnaire**

This survey is for research purposes and your anonymous answers will be used in a research study evaluating physician views on cortisone (technically known as “corticosteroid”) injections. Your participation is voluntary and will not change the care you receive.

**1. Have you ever given a patient a cortisone injection before in practice (not in training)?**

- Yes

- No

**2. If yes, what joints have you injected (check all that apply)?**

- Hip

- Knee

- Ankle

- Shoulder

- Elbow

- Wrist

- Hand

- Spine

- Never given a cortisone injection

**3. Do you think cortisone injections are safe?**

- Yes

- No

- Depends on dosage and frequency

- I don’t know

**4. How many times can you give a cortisone injection for the same problem in the same area?**

- 1 time

- 2 times

- 3 times

- No particular limit, depends on dosage and frequency

- I don’t know

**5. What best describes your understanding of the mechanism of action for cortisone?**

- It is a joint lubricant or cushion

- It anesthetizes the joint

- It is an anti-inflammatory

- I don’t know

**6. What do you consider to be adverse effects of cortisone?**

- It can injure cartilage in the joint

- It can injure ligaments around the joint

- It can decrease bone density around the joint

- It is not harmful

- I don’t know

**7. Have you ever had a cortisone injection yourself before for a joint condition?**

- Yes

- No, because I never needed one but would be open to it if offered

- No, because I was offered and refused

**8. If yes, what did you receive cortisone injection for? Select all that apply.**

- Hip

- Knee

- Ankle

- Shoulder

- Elbow

- Wrist

- Hand

- Spine

- Never received one

**9. If yes, what best describes your experience with it?**

- It worked and symptoms did not recur

- It worked for an extended period of time (more than 3 months) than symptoms recurred

- It worked for a brief period of time (less than 3 months) than symptoms recurred

- It did not help my joint symptoms

**10. What is your age?**

- 18-29

- 30-49

- 50-69

- 70-89

- Above 90

**11. What is your gender?**

- Female

- Male

- Other

- Rather Not Say

**12. What best describes your primary specialty?**

- (drop box menu of specialties)
