# Supplementary material for: Patient perceptions and knowledge of corticosteroid injections: A cross-sectional survey study
Source: PLoS One. 2026 Mar 18;21(3):e0344201. doi: 10.1371/journal.pone.0344201 (PMC12998833; doi:10.1371/journal.pone.0344201)
Supplement: S2 File — Survey instrument used to evaluate patient awareness, understanding, safety perceptions, and willingness to receive cortisone injections. (DOCX) [file pone.0344201.s002.docx]

**Survey of Patient Perception of “Cortisone” Injections Questionnaire**

This survey is for research purposes and your anonymous answers will be used in a research study evaluating patient views on cortisone (technically known as “corticosteroid”) injections. Your participation is voluntary and will not change the care you receive.

**1. Have you heard of cortisone injections before?**

- Yes

- No

**2. Assuming you have heard of cortisone injections, from whom did you first hear about them?**

- Family or friends

- Primary Care Physician

- Other physicians

- Orthopedic surgeons

- Media

- Never heard of them

**3. Have you ever had a cortisone injection before?**

- Yes

- No

**4. If yes, what did you receive cortisone injection for? Select all that apply.**

- Hip

- Knee

- Shoulder

- Spine

- Wrist

- Hand

- Never received one

**5. If you have been treated with cortisone injections before, is your physician good at describing its pharmacological, therapeutic, and side effects?**

- Always explained

- Usually explained

- Explained only when the patient asks

- Not explained

- Never received one

**6. From your understanding, how many times can you receive a cortisone injection for the same problem in the same area?**

- 1 time

- 2 times

- 3 times

- No particular limit, depends on dosage and frequency

- I don’t know

**7. What best describes your understanding of cortisone?**

- It masks pain

- It numbs pain

- It is an anti-inflammatory

- I don’t know

**8. Do you think cortisone injections are safe?**

- Yes

- No

- Depends on dosage and frequency

- I don’t know

**9. If you think it is unsafe, what do you consider is harmful about cortisone?**

- It can injure cartilage in the joint

- It can injure ligaments around the joint

- It can decrease bone density around the joint

- It is not harmful

- I don’t know

**10. If you were told you would benefit from it, would you be willing to receive a cortisone injection?**

- Yes

- No

- I don’t know

**11. What is your age?**

- 18-29

- 30-49

- 50-69

- 70-89

- Above 90

**12. What is your gender?**

- Female

- Male

- Other

- Rather Not Say

**13. What is your highest level of education?**

- Never finished High School

- High School

- Vocational Training

- College

- Graduate School

- Professional School
